# Correlative light and immuno-electron microscopy of retinal tissue cryostat sections

**DOI:** 10.1371/journal.pone.0191048

**Published:** 2018-01-09

**Authors:** Thomas Burgoyne, Amelia Lane, William E. Laughlin, Michael E. Cheetham, Clare E. Futter

**Affiliations:** 1 Institute of Ophthalmology, University College London, London, United Kingdom; 2 Primary Ciliary Dyskinesia Service, Electron Microscopy Unit, Department of Paediatrics, Royal Brompton Hospital, Sydney Street, London, United Kingdom; University of Florida, UNITED STATES

## Abstract

Correlative light-electron microscopy (CLEM) is a powerful technique allowing localisation of specific macromolecules within fluorescence microscopy (FM) images to be mapped onto corresponding high-resolution electron microscopy (EM) images. Existing methods are applicable to limited sample types and are technically challenging. Here we describe novel methods to perform CLEM and immuno-electron microscopy (iEM) on cryostat sections utilising the popular FM embedding solution, optimal cutting temperature (OCT) compound. Utilising these approaches, we have (i) identified the same phagosomes by FM and EM in the retinal pigment epithelium (RPE) of retinal tissue (ii) shown the correct localisation of rhodopsin on photoreceptor outer segment disc like-structures in iPSC derived optic cups and (iii) identified a novel interaction between peroxisomes and melanosomes as well as phagosomes in the RPE. These data show that cryostat sections allow easy characterisation of target macromolecule localisation within tissue samples, thus providing a substantial improvement over many conventional methods that are limited to cultured cells. As OCT embedding is routinely used for FM this provides an easily accessible and robust method for further analysis of existing samples by high resolution EM.

## Introduction

The localisation of macromolecules and the spatial mapping of their interactions is key to understanding how cellular signalling is regulated and how it can become dysregulated in disease. The most accessible methodology for subcellular localisation for most researchers employs immunofluorescence microscopy (iFM). Although the effective resolution of fluorescence microscopy has been enhanced by the development of super-resolution methods it is still far below that achievable by the more technically demanding immuno-electron microscopy (iEM). With both methodologies, macromolecules are typically labelled with antibodies conjugated to a fluorophore (for iFM) or gold particle (for iEM). There are multiple advantages and disadvantages of both methodologies. EM allows the location of macromolecules to be visualised in the context of all subcellular organelles, which can be identified on the basis of their ultrastructure (staining all cellular membranes with heavy metals). In contrast FM relies on the presence of additional dyes/fluorescent probes to identify subcellular organelles. FM potentially allows a far greater cell/tissue volume to be analysed, since EM relies typically on ≤100nm sections, which must be searched at high magnification to identify 5-15nm diameter gold particles. Sensitivity of labelling tends to be lower for EM and the various embedding and sectioning methods to preserve antigenicity of the specimen involve compromise in cell/tissue preservation and can require highly specialised equipment. An increasing range of dyes and fluorescent probes have been developed for FM that allow the highly sensitive detection of proteins, lipids and protein:protein interactions, but many of these are unsuitable for EM.

CLEM makes it possible to take advantage of both methodologies and allows detection of very small quantities of the target macromolecule in relatively large specimen volumes by FM that can then be located by EM, either with or without additional iEM staining [1–5]. Existing CLEM protocols can be quite technically challenging and many are only applicable to cultured cells. Here we describe a novel CLEM method that is particularly suitable for CLEM of tissue samples. Optimal cutting temperature (OCT) is a popular tissue/cell embedding medium used for FM by many laboratories as it provides excellent sample preservation, little background fluorescence and specimens can be stored frozen for an extended period of time at -80°C. Using the method described here OCT embedded cryostat samples can be sectioned using a cryostat and prepared for EM, achieving high quality specimen preservation that allows ultrastructural analysis. Due to the widespread use of OCT in conjunction with FM this method potentially allows CLEM and conventional EM to be performed on the many tissue/cell samples already available in storage without the need to generate fresh specimens. As tissue cannot be readily permeabilised and labelled for iEM due to the limited penetration of reagents, normally ultrathin sections are labelled following either technically demanding cryo-ultramicrotomy or room temperature ultramicrotomy of LR white/Lowicryl type embedded specimens with accompanying loss of membrane preservation. Cryostat sections are ideal for tissue iEM, as they are straightforward to prepare and here we show that they can be permeabilised while maintaining good ultrastructural preservation for EM.

To test the CLEM methodology, we first determined whether the same organelle could be identified by FM and EM in sections of retinal tissue. As part of a daily process that is essential for maintaining retinal health, the distal tips of photoreceptor outer segments (OS) are shed and then phagocytosed by the closely apposed retinal pigment epithelial (RPE) cell layer [6]. The OS is the light sensing part of the photoreceptor, and rod OS consists of arrays of stacked discs enriched in the light detecting machinery, including rhodopsin. Some of this machinery is recycled from the phagosomes in the RPE back to the photoreceptor to be integrated into newly formed OS discs, while the remaining contents are retained in the phagosome during sequential stages of phagosome maturation, which culminate in fusion with lysosomes [7]. Defects in uptake of phagosomes or the maturation process are associated with a number of inherited retinal degenerative diseases [8–10]. Although cultured RPE phagocytose and degrade OS, determining how these processes breakdown in disease requires them to be studied in the context of a multi-layered tissue. Thus the ability to do CLEM on retinal tissue would aid elucidation of the molecular mechanisms that underly retinal degenerative diseases.

To further illustrate the CLEM methodology and include dual FM and iEM labelling, we examined the localisation of rhodopsin in induced pluripotent stem cell (iPSC) derived optic cups. These 3D tissue-like cultures are self-organised structures that develop a laminated organisation with all the major retinal cell types, resembling the human retina, and providing an effective *in vitro* model to study photoreceptor physiology and disease [11]. Even though they are morphologically similar, optic cup photoreceptors differ from the *in vivo* equivalent as they have a shorter less developed OS. This could be due to the lack of RPE and other intraocular tissue [12], but could also result from defective rhodopsin transport to the OS as rhodopsin knockout mice have short OS [13]. In healthy retinal tissue, rhodopsin is synthesised in the photoreceptor inner segment and transported to the OS via the connecting cilium, where it is highly concentrated on disc membranes [14]. The use of CLEM to determine whether rhodopsin is correctly localised to OS disc membrane would give greater validity to optic cups as a model to study photoreceptor biology.

Finally, we used a non-correlative approach to examine the localisation of peroxisomes in the RPE of retinal tissue. As highly abundant organelles in the RPE, peroxisomes have been implicated in general lipid homeostasis and oxidative degradation of long-chain fatty acids from phagocytosed photoreceptor OS [15,16]. How fatty acids are transferred from phagosomes to peroxisomes is not well understood. Lipid transfer between organelles can occur at membrane contact sites, regions of close membrane apposition between organelles [17–24], suggesting the possibility that membrane contact sites between peroxisomes and phagosomes would provide a platform for direct fatty acid transfer for degradation. Membrane contact sites can only be unequivocally identified by EM but peroxisomes are difficult to identify on the basis of morphology alone. The use of iEM of cryostat sections to identify peroxisomes would allow their distribution in the RPE of retinal tissue to be determined and potential contacts with other organelles to be identified.

## Materials and methods

### Mouse eyes

The mouse eyes used were existing OCT-embedded samples from five-month-old wild type C57Bl6 untreated mice that had been sacrificed by cervical dislocation at a Schedule 1-approved designated establishment in accordance with Animals (Scientific Procedures) Act 1986 (United Kingdom) and Home Office (United Kingdom) guidance rules, adhering to the Association for Research in Vision and Ophthalmology Statement for the Use of Animals in Ophthalmic and Vision Research.

### Optic cups

Control BJ inducible pluripotent stem cells (iPSC) were differentiated into photoreceptor optic cups as described [11]. Four optic cups were mounted in OCT compound, and from the cryostat sections cut, four were used in this study.

### Preparing cryostat sections

We used whole eyes from mice sacrificed 1.5 hours (2 eyes) or 5.5 hours (one eye) after light onset or optic cups, all of which had previously been fixed immediately after extraction in 4% paraformaldehyde (PFA) in PBS for 1 hour at room temperature before infusing with 30% sucrose (cyroprotectant) in PBS overnight at 4°C. Samples had then been embedded in OCT compound and frozen using a bath of acetone cooled to -78°C using dry ice. 5–20 um sections of the frozen samples were cut at -20°C with a microtome blade and placed onto slides using a cyrostat. If sections were not used straightaway they were stored at -80°C.

### Immunolabelling and confocal microscopy

Cyrostat sections were permeabilised using either 0.004% digitonin or 0.02% saponin (0.05% triton was also found to work but data is not shown) in 1% BSA in PBS for 30 mins at room temperature followed by incubating in blocking solution consisting of 1% BSA and 0.1% acetylated BSA in PBS for 30 mins at room temperature. Antibody labelling was performed by incubated the sections with antibodies against rhodopsin (mouse monoclonal from Abcam ab3267/ AB_303655 used at 1 in 500), laminin-1 (rabbit polyclonal from ThermoFisher Scientific PA1-30605/ AB_1957532 used at 1 in 200) and PMP70 (rabbit polyclonal from Novus Biologicals NBP1-87258 /AB_11009460 used at 1 in 100) in blocking solution overnight at 4°C. Secondary antibodies bound to an Alexa Fluor dye (ThermoFisher Scientific used at 1 in 500), FluoroNanogold (goat anti-rabbit from Nanoprobes 7204 used at 1 in 100) or Nanogold (goat anti-mouse from Nanoprobes 2002/AB_2637031 used at 1 in 200) in addition to fluorescent phalloidin (abcam ab176759 used at 1 in 500) in blocking solution were added to the sections for 2 hrs at room temperature. In between all antibody labelling steps, the sections were washed three times in blocking solution for 30 mins at room temperature on a rocking platform. Sections immunolabelled with a fluorescent secondary antibody were mounted in PBS (instead of mounting media as it is easier to remove the coverslip after FM imaging) and visualised for FM using a Zeiss 700 confocal and high and low magnification images were taken at regions of interest.

### Electron microscopy preparation

**Fig 1:**
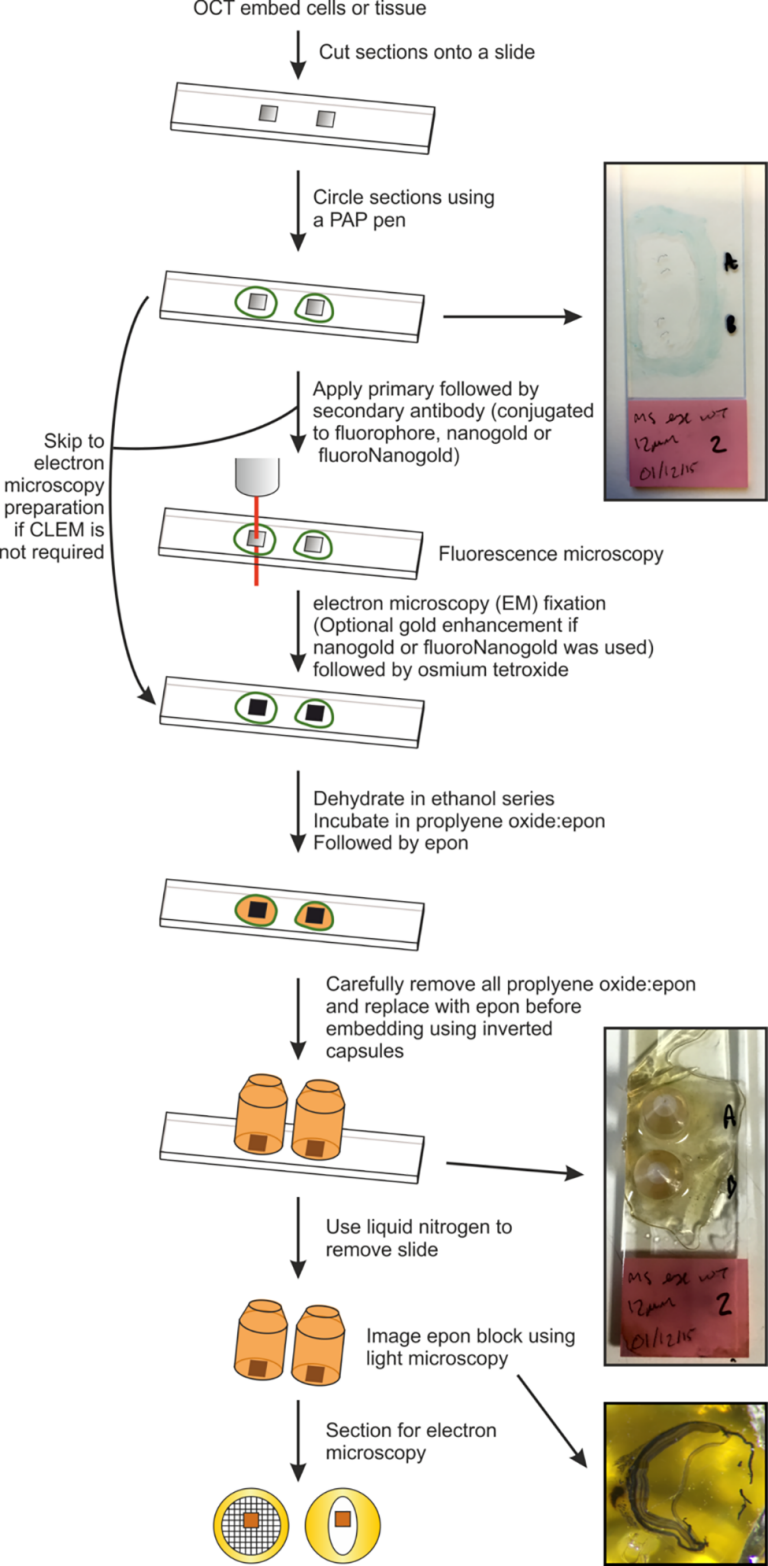
Outline method for correlative light and electron microscopy of cryostat sections.

Cyrostat sections were re-fixed in 2% PFA, 2% glutaraldehyde in 0.1M cacodylate buffer for 1 hr at room temperature. If Nanogold or FluoroNanogold secondary antibodies were used the sections were incubated with a gold enhance solution which was prepared in accordance with manufacturers specifications (Nanoprobes) on ice at 4°C. The sections were incubated in 1% osmium tetroxide, 1.5% potassium ferrocyanide in distilled water for 1 hr in the dark at 4°C. To provide extra contrast to the sections they were incubated in aqueous uranyl acetate for 1hr at room temperature. The sections were dehydrated in increasing concentration of ethanol (70%, 90% and 100%) and in a mixture of propylene oxide:epon (1:1) for 30 mins at room temperature. All the propylene oxide:epon was carefully removed by applying four changes of epon ever 20 mins at room temperature before embedding in epon overnight at 60°C. 100 nm sections were cut and examined on a JEOL 1010 EM. An outline of the method is shown in Fig 1.

### Image software

Microsoft Image Composite Editor (ICE) was used to generate montages from low magnification FM and EM images and Photoshop was used to overlay the EM on top of FM images. ImageJ was used to quantify the number of melanosomes or phagosomes in contact with peroxisomes when apposing membranes were within 30 nm of each other.

## Results

**Fig 2:**
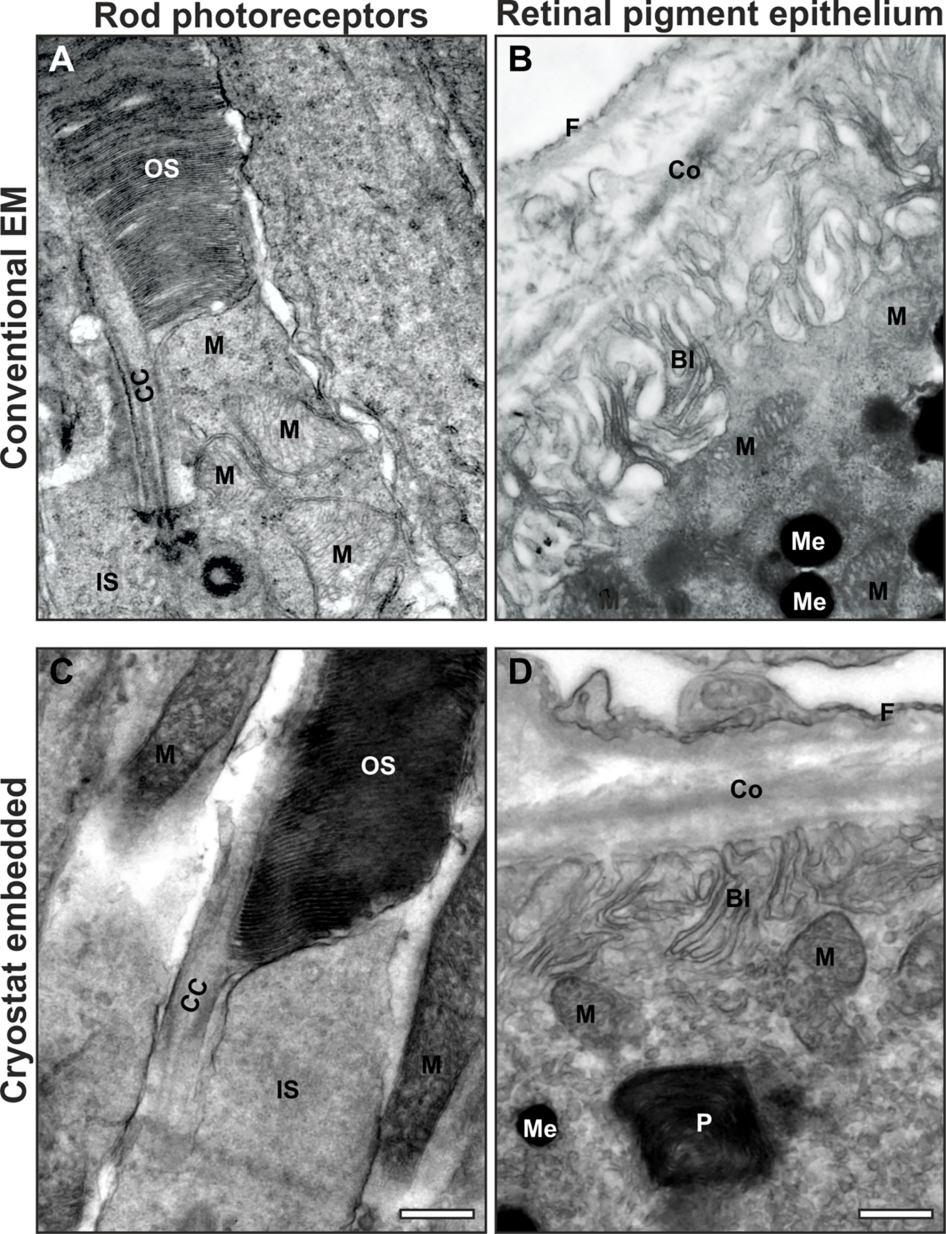
Mouse retinal tissue prepared conventionally and from OCT embedded sections for electron microscopy. (A & B) Conventional fixed tissue and (C & D) OCT sections of tissue prepared for EM show little difference in the preservation quality. Organelles including mitochondria (M), melanosomes (Me) and phagosomes (P), in addition to membranes such as basal infoldings (BI) and photoreceptor outer segment (OS) discs and structural features such as collagen in Bruchs membrane (Co) and fenestrae of choroidal endothelial cells (F) are well preserved. (A & C) the connecting cilium is indicated by CC and the photoreceptor inner segment IS. Scale bar = 500nm.

The mouse eyecup is a multi-layered tissue up to 0.5mm thick and so its preservation and infiltration with embedding reagents used for conventional EM can be challenging. In this study, we found that 10–50 μm cryostat sections of OCT embedded mouse retinal tissue could be embedded for conventional EM with a high degree of preservation (Fig 2). Organelles including mitochondria and melanosomes had very similar profiles to those in samples prepared using conventional EM techniques. In addition, membranes remained intact and photoreceptor OS discs, RPE basal infoldings and fenestrated endothelia were clearly preserved.

**Fig 3:**
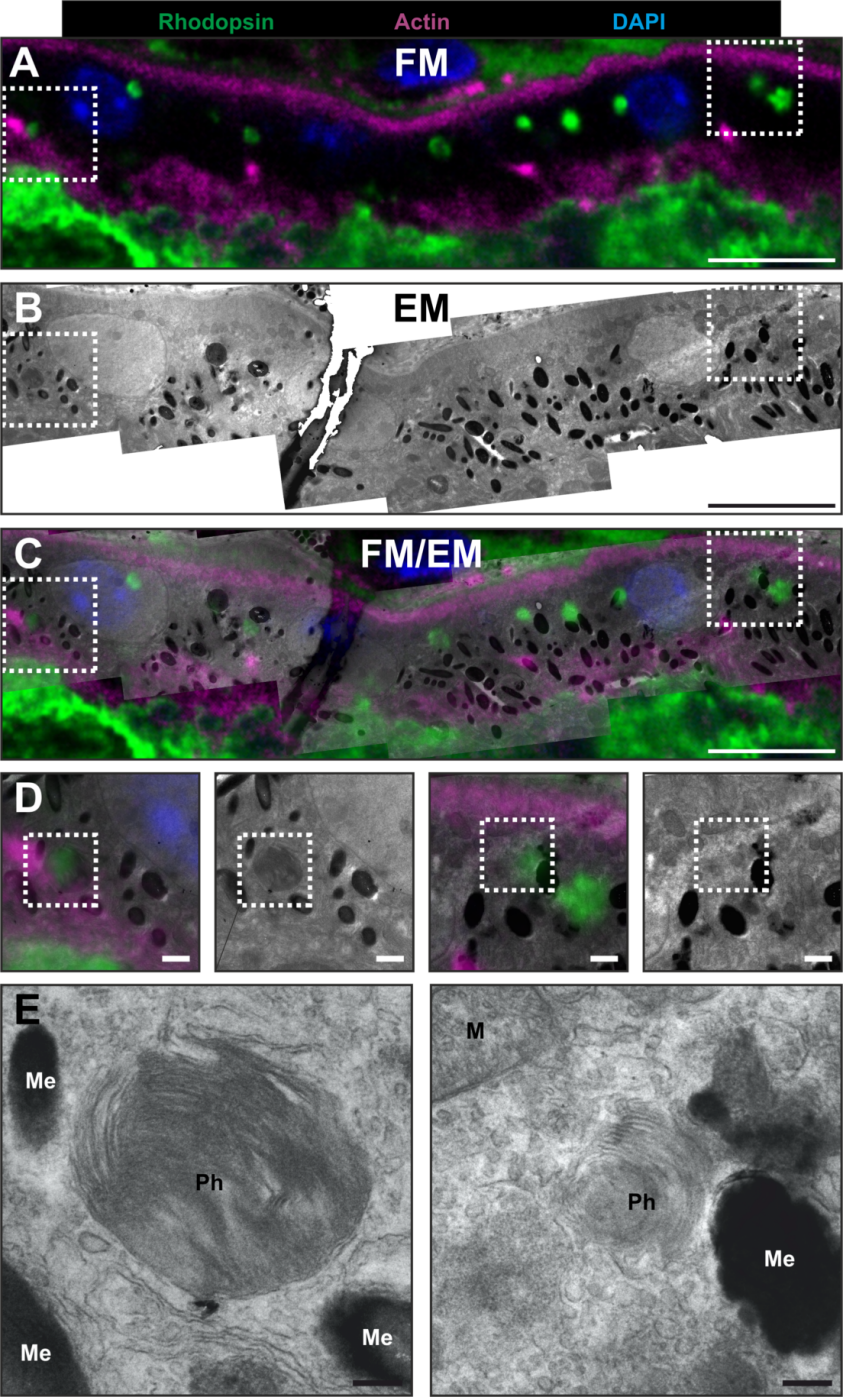
Correlative light electron and microscopy of cryostat sections can be used to identify rhodopsin enriched phagosomes in the retinal pigment epithelium (RPE) cell layer. The same region of RPE viewed by (A) fluorescence microscopy (FM) and (B) electron microscopy (EM), with an overlay of the two in (C). The boxed regions in (A-C) are shown at higher magnification in (D) highlighting regions that include FM rhodopsin staining (green) overlapping with phagosomes seen by EM. (E) Higher magnification of phagosomes (Ph) boxed in (D), surrounded by melanosomes (Me) and mitochondria (M). Scale bar = (A-C)– 10um (D)– 1um (E)– 250nm.

Following the method outlined in Fig 1, FM of cryostat sections stained with selected primary and fluorescent secondary antibodies allows regions of interest to be identified and subsequently mapped onto EM data (Fig 3). Before embedding for EM, cryostat sections of mouse retina were stained with fluorescent phalloidin to stain actin filamants, DAPI for nuclei and specific primary anti-rhodopsin followed by fluorescent secondary antibody and imaged by FM. Low magnification FM images were montaged to generate an overview of each sample so that after embedding the section for EM, the montage could be overlaid onto light microscopy images of the cryostat section within the resin EM block (S1A–S1C Fig and S2A–S2C Fig). This allowed a region of interest to be selected and allowed the identification of the part of the sample blockface to be sectioned. By comparing features between the high magnification FM and EM data, images were correlated and overlaid (Fig 3A–3C, S1D–S1F and S2 Fig). Within the overlay the rhodopsin labelling in the RPE overlapped with phagosomes (Fig 3D) that are morphologically identifiable by EM (Fig 3E). This approach also allowed the identification of retinal blood vessels in retinal tissue using phalloidin and anti-Laminin-1 (asterisks in S2G–S2I).

**Fig 4:**
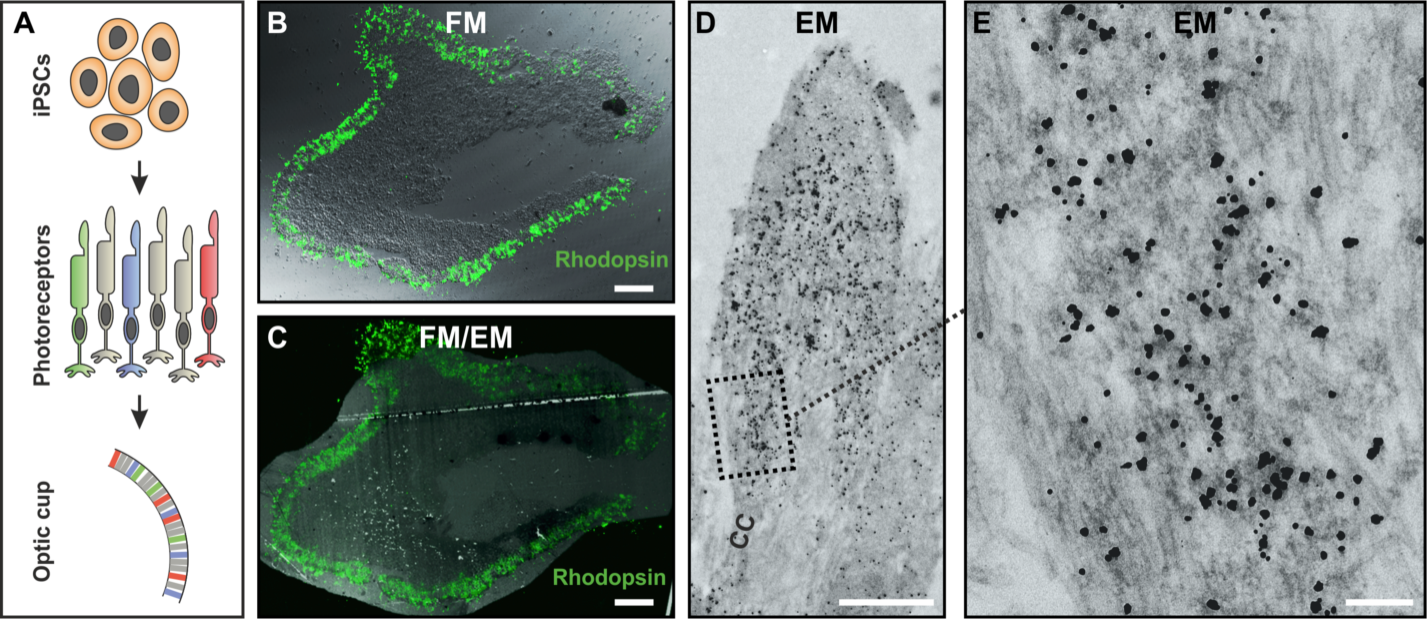
Correlative light and electron microscopy of cryostat sections of control iPSC optic cup using dual fluorescence microscopy (FM) and nano-gold rhodopsin labelling. (A) Diagram outlining the differentiation of inducible pluripotent stem cells (iPSCs) into optic cups with photoreceptors. (B) Confocal FM overlay on top of differential interference contrast (DIC) image (C) FM overlay on top of electron microscopy (EM) image. (D) EM with (E) a high magnification region showing concentrated rhodopsin localisation in the photoreceptor OS region of the optic cups. The connecting cilia in (D) is indicated by CC. Scale bar = (B & C) 100um, (D) 2um and (E) 200nm.

As phagosomes and blood vessels are relatively large compared to a single macromolecule, IF staining of these structures can be mapped onto EM images without the need for immuno-gold labelling. When examining macromolecules that are dispersed throughout a cell or localised to small structures, dual FM and EM immunolabelling may be required. The recent development of secondary antibodies conjugated to both a fluorophore and a nanogold particle allows this, as the same primary antibody molecule can be imaged by both FM and EM. Cryostat sections of optic cups derived from iPSC (Fig 4A) were stained with anti-rhodopsin antibody followed by labelling with a fluorophore/gold secondary antibody. After imaging by FM (Fig 4B) sections were embedded for EM, including a gold enhancement step to allow visualisation of the gold particles by EM (Fig 4C–4E). The FM and FM/EM combined image show intense rhodopsin immunoreactivity was localised to the distal portion of the rod photoreceptor (Fig 4B and 4C). A closer inspection by EM allowed rhodopsin localisation to be confirmed by the gold labelling on disc membranes distal to the connecting cilium (CC) within the OS region of the optic cups (Fig 4D and 4E).

**Fig 5:**
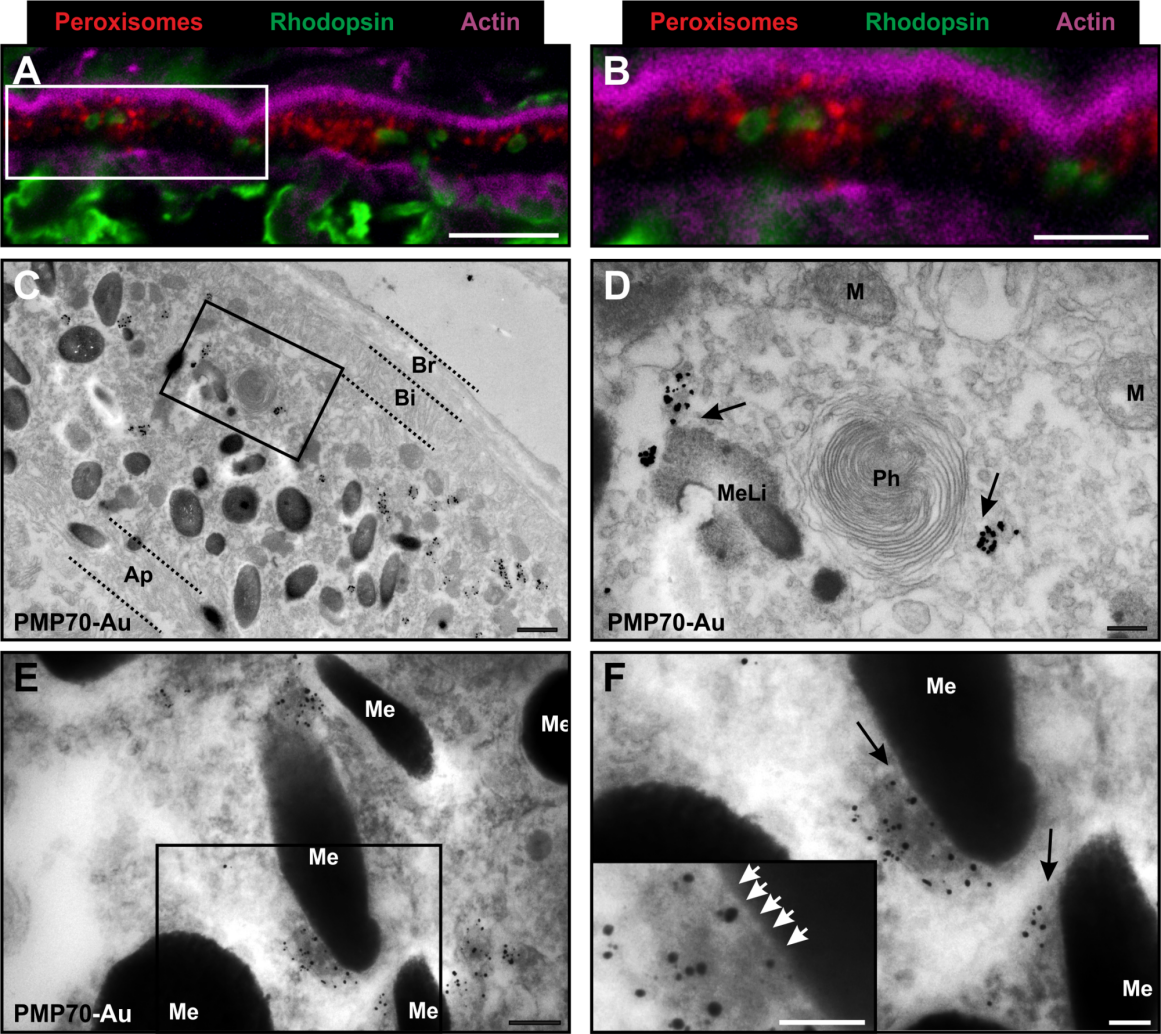
Immuno-electron microscopy (iEM) labelling of cryostat sections allows the identification of peroxisomes in the RPE, illustrating the close contact with phagosomes and melanosomes. (A-B) FM images showing the localisation of peroxisomes (red) around rhodopsin enriched phagosomes (green) in the RPE cell layer. (C) Low magnification electron microscopy image of RPE cell layer with gold labelled peroxisomes that are localised towards the basal cell surface close to the basal infolding (Bi) and Bruch’s membrane (Br) and away from the apical processes (Ap). The box highlights a region with a phagosome (Ph) that is shown at higher magnification in (D) with a gold labelled peroxisome in close proximity and other organelles nearby, including melanolipofuscin granules (MeLi) and mitochondria (M). (E) Gold labelled peroxisomes in contact with melanosomes (Me) with a high magnification image (F). Contacts between the peroxisomes and melanosomes (black arrows) and in the zoomed insert tethers between the organelles can be seen (white arrows) Scale = (A)– 10um (B)– 5um (C)– 1um (D-E)– 250nm (F)– 100nm.

As a preliminary investigation of peroxisomes in the RPE, eyes that had been extracted from a mouse at 9.30 am and from a second mouse at 1.30 pm were examined. As peroxisomes are so numerous in the RPE, to examine them in detail CLEM analysis was not required, instead non-correlative IF and iEM was used. Peroxisomes were labelled using an antibody against the 70-kDa peroxisomal membrane protein (PMP70), and IF indicated staining clustered around phagosomes labelled with an anti-rhodopsin antibody (Fig 5A and 5B). Performing iEM on cryostat sections allowed confirmation that PMP70 staining localised exclusively to peroxisomes and allowed the subcellular distribution of these organelles to be examined. As has been previously reported peroxisomes were situated mostly towards the basal area, and almost entirely excluded from the apical region of RPE cells (Fig 5C and S3 Fig) [25]. Additionally, a proportion of peroxisomes clearly made contact with phagosomes (Fig 5C and 5D and S4 Fig) and we also observed many peroxisomes in contact with melanosomes and mitochondria (Fig 5E and 5F and S5 Fig). When examining the eye extracted at 9.30 am 11.4% of melanosomes (n = 490) were observed in contact with peroxisomes, whereas at 1.30 pm there was a reduced proportion of 7.3% melanosome (n = 372) in contact with peroxisomes. In contrast a decreased number of phagosomes were observed interacting with peroxisomes at 9.30 am compared to 1.30 pm, with 34.9% (n = 43) and 43.2% (n = 44) respectively.

## Discussion

We present a new method of sample preparation that allows CLEM and iEM analysis of samples that were originally prepared for FM. Using this approach the specimen preservation is almost equivalent to tissue prepared using conventional methods for EM analysis. Therefore, this approach could be extremely valuable to many laboratories, as it can save time and money by minimising sample preparation as well as allowing contemporary EM analysis of old or unique cryostat samples. Additionally, most of the current CLEM methodologies are better suited for examining macromolecules in cultured cells, whereas cryostat sections are ideal for performing CLEM on tissue samples. We found that to obtain reproducibly good EM preservation and antibody labelling of cryostat samples it was important to use good quality cryostat sections, high affinity antibodies, adequate sample washing in between incubations and careful removal of all propylene oxide before embedding (as outlined in the methods section and Fig 1). Within a week from initial fixation, specimens can be analysed by FM and embedded and sectioned for EM. With an additional few days required to examine multiple EM sections and find the region of interest, this is comparable to standard CLEM methods that involve culturing cells on gridded coverslips as the imaging and processing times are very similar [26]. The iEM approach using cryostat sections can take up to a day or two longer than for cryo-ultramicrotomy due to the sample processing time but the timeframe is similar to room temperature ultramicrotomy of LR white/Lowicryl type embedded specimens [27,28].

In our first example, as a proof of principle of the CLEM methodology, we were able to identify the same phagosomes in the RPE by IF and EM. To achieve this, IF rhodopsin labelling alone of phagosomes was inadequate to align and overlay IF and EM images, and therefore additional IF labelling to visualise key features was required. These included DAPI for the nuclei and actin to detect the apical and basal surfaces of the RPE. Without these additional markers, phagosome staining alone would not suffice, as within a single IF z-slice (~ 1 μm) many labelled phagosomes are not present in a 100nm EM section of the same region. Once matched together, the IF gave precise positioning of phagosomes as confirmed by high magnification EM images of the morphologically dense membranous phagosome structures. As rhodopsin staining and markers involved in phagosome maturation in the RPE can be readily identified by IF and further assessed in detail at the high resolution achievable by EM, this approach could be highly beneficial for future studies. These studies include examining phagosome maturation in greater detail in human tissue samples and retinal degenerative disease models to determine how this process can become defective and lead to retinal disease [29,30].

Our knowledge of the molecular regulation of photoreceptor OS biogenesis and renewal is incomplete, partly because of the lack of adequate photoreceptor culture systems. Therefore, the development of optic cups from stem cells potentially represents a major advance, provided that membrane traffic systems leading to OS biogenesis are functional. The FM rhodopsin staining when overlaid onto the differential interference contrast (DIC) and low magnification EM images indicate the most intense immunoreactivity at the outer region of the optic cups. By immuno-labelling with a secondary antibody bound to both a fluorophore and a gold particle to allow iEM, in addition to CLEM, we were able to show that rhodopsin localises to disc like structures in the rudimentary OS of the optic cup photoreceptors, similar to its localisation to OS discs *in vivo*. This helps to validate the optic cups as a good model for analysing the molecular regulation of photoreceptor OS biogenesis and renewal, defects in which underlie multiple retinal degenerations [31–33].

Using a non-correlative imaging approach, we have provided potential new insight into peroxisome-organelle interactions in the RPE. FM labelling of peroxisomes indicates that they surround basally situated phagosomes in the RPE, and when examining this positioning more closely by iEM, many of the phagosomes were in contact with at least one peroxisome. Phagosome maturation proceeds in sequential stages as they move through the RPE towards the basal surface where lysosomal fusion takes place and OS discs are lost. As peroxisomes are almost entirely absent from the apical half of the RPE the peroxisome:phagosome interactions must occur at a late stage in the maturation process. Even as a preliminary investigation, more phagosomes interacting with peroxisomes were observed at 1.30pm (when more mature phagosomes are close to the basal surface [7]) compared to 9.30am. The presence of recognisable OS discs within the phagosome indicates that peroxisomes interact with phagosomes shortly before phagosomes fuse with lysosomes. This interaction could be the key platform for direct transfer of long-chain fatty acids to peroxisomes for oxidative degradation before the phagosomes are degraded. Interestingly, we observed many melanosomes interacting with peroxisomes which, to our knowledge, has not been reported before. As melanosomes frequently interact with phagosomes in the RPE [7], it is possible the peroxisomes influence phagosome maturation indirectly through melanosome interactions. This may explain the increase in peroxisome:melanosome interactions at 9.30 am compared to 1.30 pm, but as a preliminary study this needs to be investigated further. Lastly, we observed peroxisome:mitochondria interactions that could reflect the role of mitochondria in peroxisome biogenesis or a shared platform for fatty acid oxidation [34]. To understand the significance of these interactions requires further work that could provide new insight into phagosome maturation and RPE function.

In the examples presented here we have used mouse retina and cultured human optic cups, but this method is potentially extendable to multiple tissue and cell samples. Combining FM and EM provides a powerful tool to accurately study macromolecule localisation, and with the advent of significantly improved confocal resolution, the benefits of using CLEM are rapidly advancing. Additionally, recent advances in cryo-EM allow samples to be high-pressure freezing before imaging by FM [35]. This allows samples to be frozen in vitreous ice providing greater preservation and achievable EM resolution over the use of aldehyde fixation methods [36,37]. Unfortunately, the equipment to perform this type of CLEM is expensive and technically demanding, but in the future as cryo-CLEM develops it may become more accessible.

## Supporting information

S1 Fig Low magnification correlative light and electron microscopy mapping of FM and EM images used to identify the region of interest in Fig 3. (A-C) Are low magnification IF images mapped onto retinal cryostat section embedded within a resin EM block. (D-F) higher magnification EM overlay onto IF data with the box in (F) highlighting the region examined in Fig 3. Scale = (A-C) 500 um, (D-E) 50um.
(TIF)

S2 Fig Correlative light and electron microscopy of an OCT section of mouse retinal tissue. (A) Fluorescence microscopy (FM) image of laminin-1 and actin (phalloidin) staining. (B) Light microscopy (LM) image of embedded section in epon resin and C) with FM overlaid on top. (D) high magnification FM image of an area of interest with (E) electron microscopy (EM) image of the same area and (F) an overlay of the FM and EM. (G–F) Higher magnification of the boxed regions highlighted in (D–F) showing blood vessels (asterisks) as visible by the laminin-1 staining in (G & I) and the EM ultrastructure in H). Scale = (A–C) 500um and (D–F) 50 um (G—I) 10 um.
(TIF)

S3 Fig Gold labelled peroxisomes are positioned between the cell medial (longest dotted line) and basal membrane in RPE cells. Peroxisomes are almost completely absent between the cell medial and apical surface. The regions of RPE include the basal infoldings (BI) and the apical processes (Ap) and just beyond the RPE basal surface is Bruch’s membrane (Br). Scale = 1um.
(TIF)

S4 Fig Further examples of gold labelled peroxisomes in contact with phagosomes (Ph) in the RPE. Other organelles include melanosomes (Me) and mitochondria (M). Scale = 250nm.
(TIF)

S5 Fig Additional examples of gold labelled peroxisomes in contact with melanosomes (Me) in the RPE. Scale = 250 nm.
(TIF)
