# Supplementary figures and images for: Correlative light and immuno-electron microscopy of retinal tissue cryostat sections
Source: PLoS One. 2018 Jan 9;13(1):e0191048. doi: 10.1371/journal.pone.0191048 (PMC5760081; doi:10.1371/journal.pone.0191048)

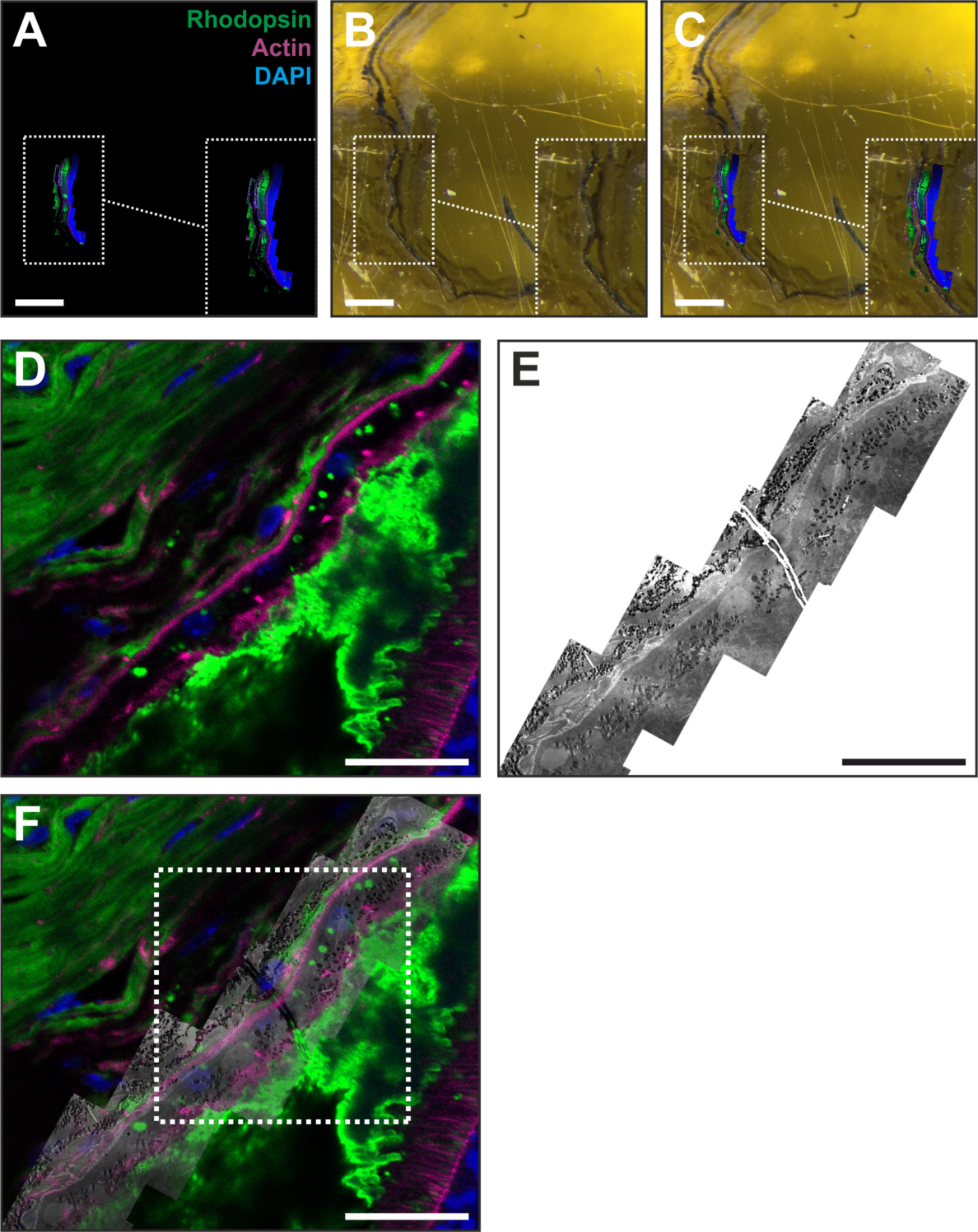

Supplement: S1 Fig — (A-C) Are low magnification IF images mapped onto retinal cryostat section embedded within a resin EM block. (D-F) higher magnification EM overlay onto IF data with the box in (F) highlighting the region examined in Fig 3. Scale = (A-C) 500 um, (D-E) 50um. (TIF) [file pone.0191048.s001.tif]

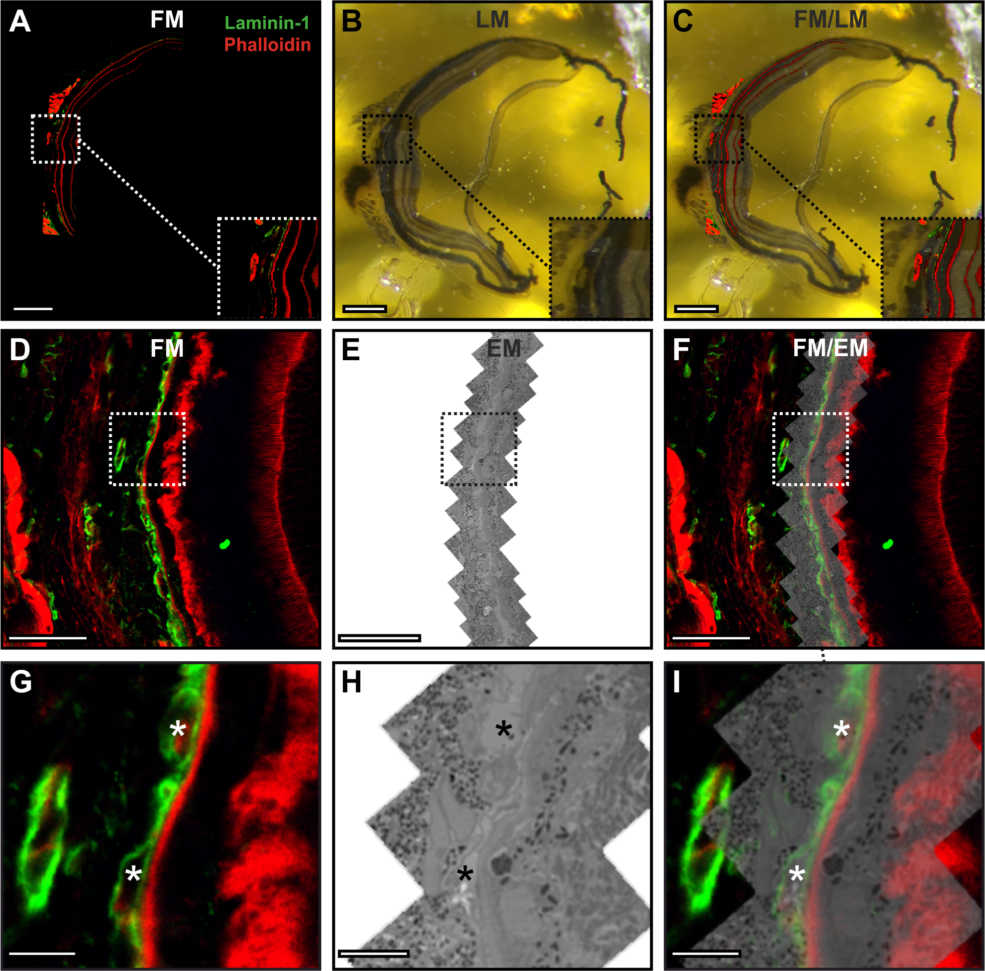

Supplement: S2 Fig — (A) Fluorescence microscopy (FM) image of laminin-1 and actin (phalloidin) staining. (B) Light microscopy (LM) image of embedded section in epon resin and C) with FM overlaid on top. (D) high magnification FM image of an area of interest with (E) electron microscopy (EM) image of the same area and (F) an overlay of the FM and EM. (G–F) Higher magnification of the boxed regions highlighted in (D–F) showing blood vessels (asterisks) as visible by the laminin-1 staining in (G & I) and the EM ultrastructure in H). Scale = (A–C) 500um and (D–F) 50 um (G—I) 10 um. (TIF) [file pone.0191048.s002.tif]

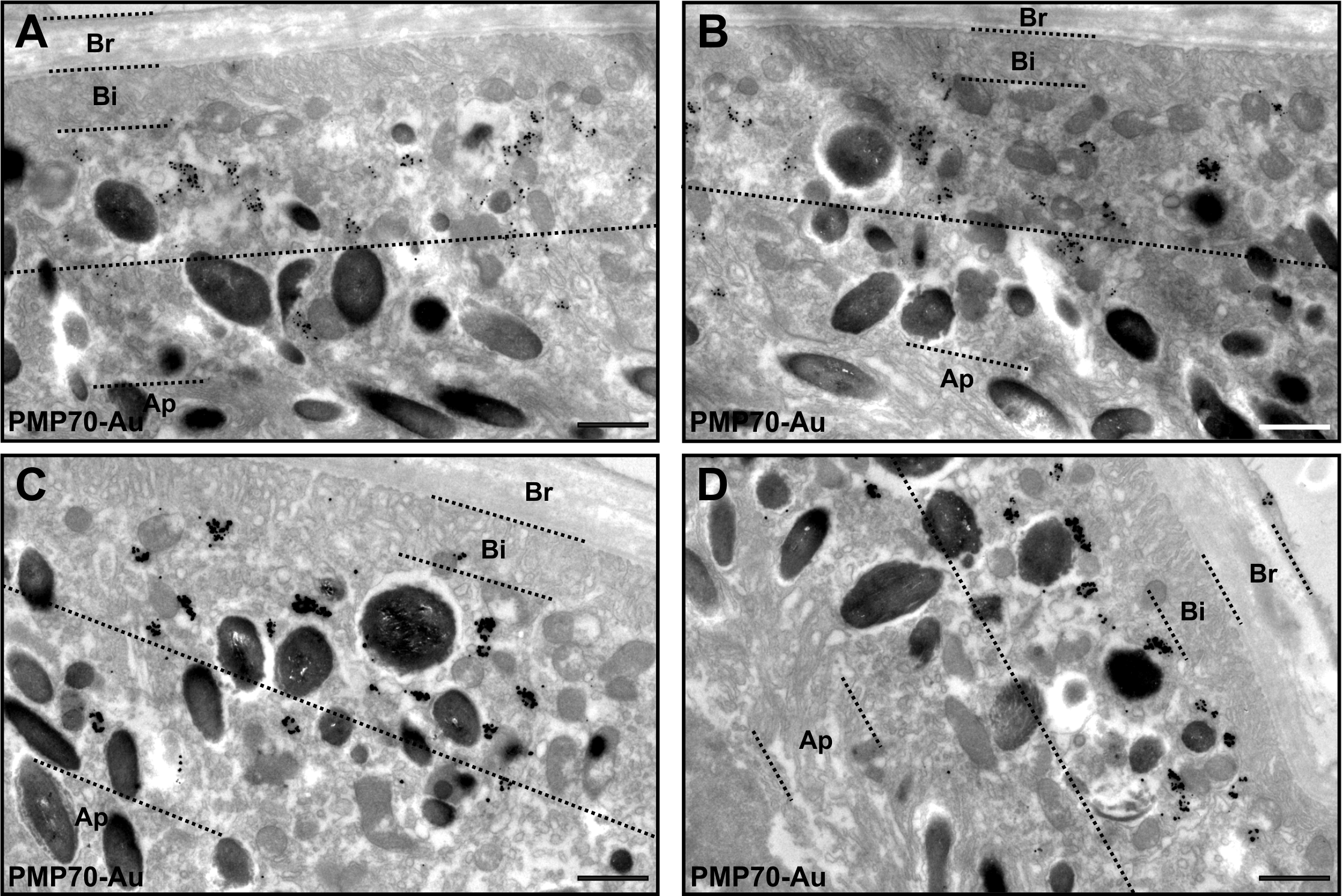

Supplement: S3 Fig — Peroxisomes are almost completely absent between the cell medial and apical surface. The regions of RPE include the basal infoldings (BI) and the apical processes (Ap) and just beyond the RPE basal surface is Bruch’s membrane (Br). Scale = 1um. (TIF) [file pone.0191048.s003.tif]

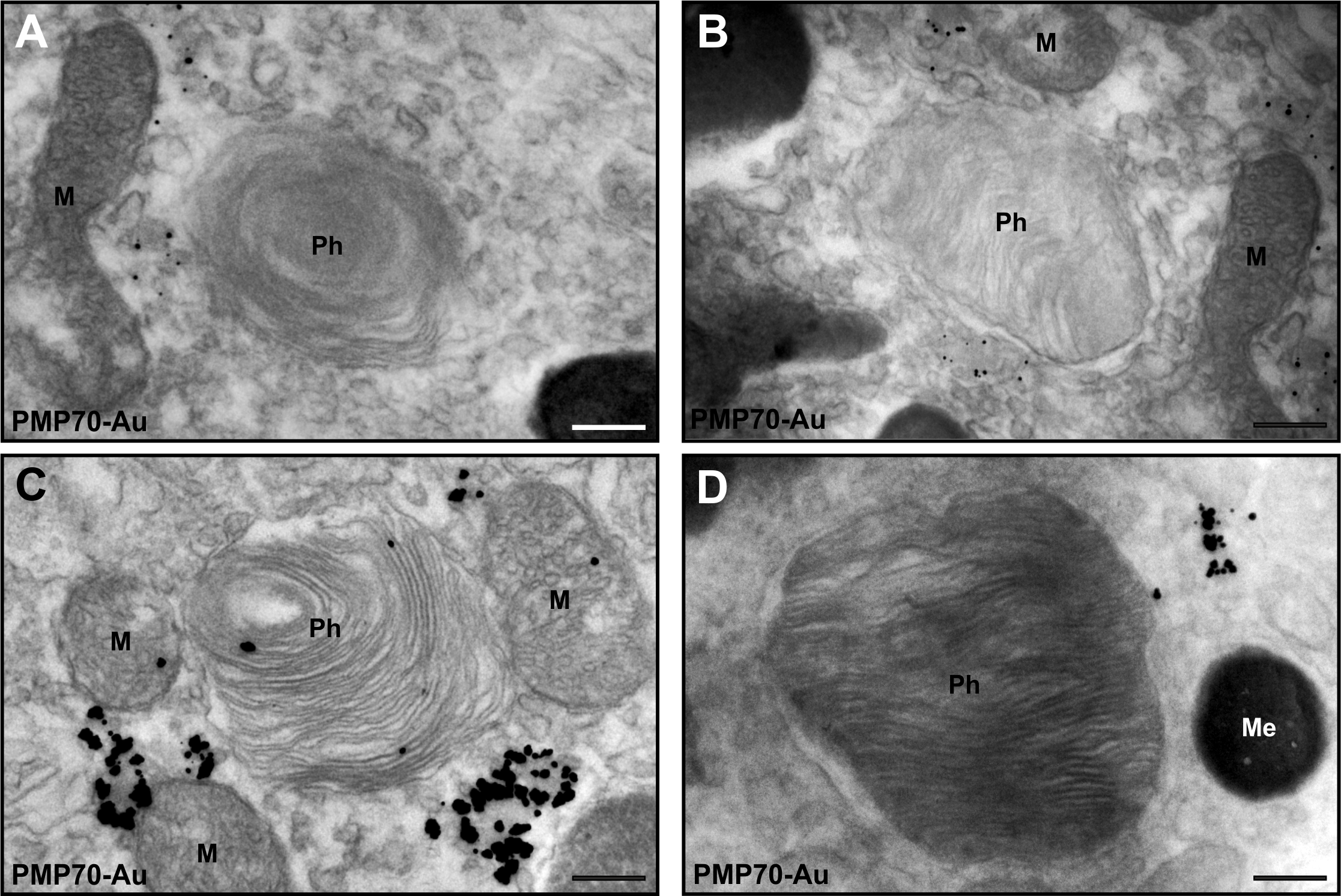

Supplement: S4 Fig — Other organelles include melanosomes (Me) and mitochondria (M). Scale = 250nm. (TIF) [file pone.0191048.s004.tif]

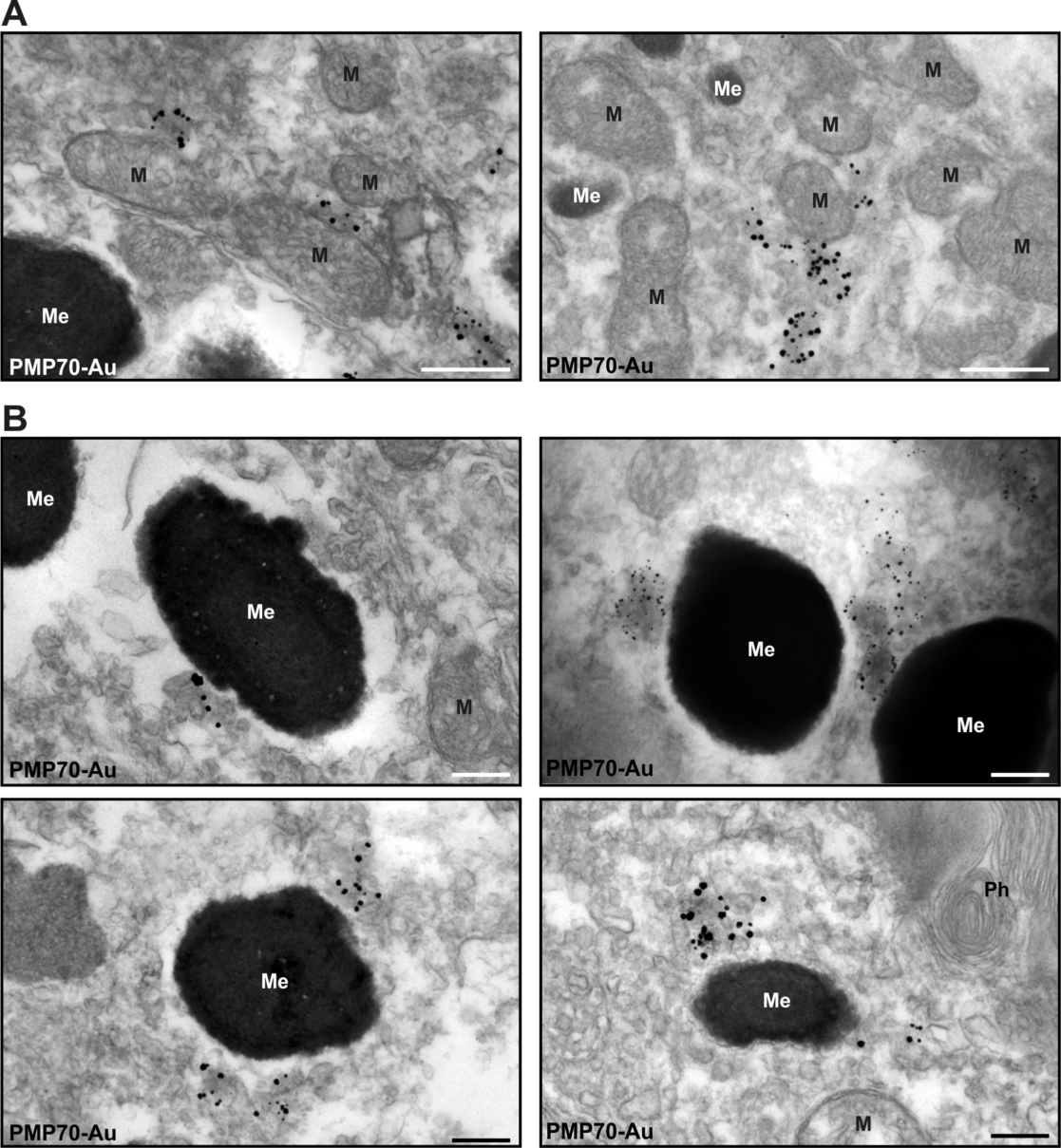

Supplement: S5 Fig — Scale = 250 nm. (TIF) [file pone.0191048.s005.tif]
